# CasHRA (Cas9-facilitated Homologous Recombination Assembly) method of constructing megabase-sized DNA

**DOI:** 10.1093/nar/gkw475

**Published:** 2016-05-24

**Authors:** Jianting Zhou, Ronghai Wu, Xiaoli Xue, Zhongjun Qin

**Affiliations:** 1 Key Laboratory of Synthetic Biology, Institute of Plant Physiology and Ecology, Shanghai Institutes for Biological Sciences, Chinese Academy of Sciences, Shanghai 200032, China; 2 University of the Chinese Academy of Sciences, Beijing 100049, China

## Abstract

Current DNA assembly methods for preparing highly purified linear subassemblies require complex and time-consuming *in vitro* manipulations that hinder their ability to construct megabase-sized DNAs (e.g. synthetic genomes). We have developed a new method designated ‘CasHRA (Cas9-facilitated Homologous Recombination Assembly)’ that directly uses large circular DNAs in a one-step *in vivo* assembly process. The large circular DNAs are co-introduced into *Saccharomyces cerevisiae* by protoplast fusion, and they are cleaved by RNA-guided Cas9 nuclease to release the linear DNA segments for subsequent assembly by the endogenous homologous recombination system. The CasHRA method allows efficient assembly of multiple large DNA segments *in vivo*; thus, this approach should be useful in the last stage of genome construction. As a proof of concept, we combined CasHRA with an upstream assembly method (Gibson procedure of genome assembly) and successfully constructed a 1.03 Mb MGE-syn1.0 (Minimal Genome of *Escherichia coli*) that contained 449 essential genes and 267 important growth genes. We expect that CasHRA will be widely used in megabase-sized genome constructions.

## INTRODUCTION

The assembly of small DNA fragments into large constructs, such as the genes involved in biochemical pathways, biological machinery, and even entire genomes, is one of the most fundamental requirements of synthetic biology. State-of-the-art *in vitro* DNA assembly methods have been developed to fulfil different purposes (1,2). Restriction endonuclease assembly methods (including BioBricks (3), BglBricks (4) and Golden gate (5)) are widely used to assemble standardized biological components, and *in vitro* overlap assembly methods (including InFusion (6), SLIC (7) and Gibson one-step isothermal assembly (8)) are useful for large DNA constructions. The efficient homologous recombination observed in *Saccharomyces cerevisiae* has greatly simplified the construction of DNA molecules up to several hundred kilobases (9,10).

The techniques required to synthesize a complete genome are important for synthetic biology. The ongoing Synthetic Yeast Genome Project (Sc2.0) has demonstrated the plasticity of the yeast genome by synthesizing chromosome arms (11). As a milestone achievement, the first heavily edited yeast chromosome, synIII (273 kb), was synthesized by 11 successive rounds of DNA assembly *in vivo* and used to replace the native chromosome III (12). This method avoided the direct assembly of large DNAs and allowed for step-by-step testing of the functionality of the newly synthesized regions. However, a similar strategy would not be suitable for genome synthesis in other organisms without an efficient endogenous homologous recombination system. Because the smallest free-living organisms have genomes larger than 1 Mb (13), the ability to synthesize a megabase-sized genome is of great importance. The first complete 1.08 Mb *Mycoplasma mycoides* genome was synthesized by three rounds of assembly in yeast following the Gibson procedure, and its functionality was demonstrated in another closely related species, *M. capricolum* (14). The Gibson procedure of genome assembly allows for the one-step assembly of more than 10 DNA fragments in yeast and has an efficiency of 10–100%. However, the assembly efficiency decreases drastically down to 1/48 at the final stage of genome assembly because this method requires a sufficient quantity of high-quality large subassembly DNA segments from complex *in vitro* manipulation procedures. All eleven 100 kb assembly intermediates were isolated from yeast, and then two steps of purification and one step of enrichment were performed to remove the host chromosomal DNA contamination (14). These difficult and time-consuming *in vitro* manipulations resulted in low efficiency.

In recent years, the RNA-guided nuclease Cas9 has become widely used in prokaryotic and eukaryotic genome editing (15–20). Guided by designed RNAs, Cas9 can introduce double-stranded DNA breakage at the defined position, which dramatically increases the efficiency of targeted genome deletions, mutations, and insertions (15). Cas9 has also been reported to facilitate direct multiplex integrations of DNA parts assembled *in vivo* in yeast (21).

To avoid the complex and time-consuming *in vitro* manipulations of large linear DNAs during genome assembly, we have developed a new method in which circular DNAs are used directly in a one-step *in vivo* assembly. We designated this method CasHRA (Cas9-facilitated Homologous Recombination Assembly). The large circular DNAs were cut by target RNA-guided Cas9 *in vivo* to release the linear DNAs for direct assembly by overlapping sequences and homologous recombination in yeast. By eliminating the guide RNAs, the assembled circular DNAs could be directly used for the next round of DNA assembly. We tested the efficiency of CasHRA in assemblies with both small and large circular DNAs. To demonstrate the utility of CasHRA for genome assembly, we combined this method with the Gibson procedure of genome assembly as an upstream assembly method to construct a 1.03 Mb MGE-syn1.0 (Minimal Genome of *E. coli*).

## MATERIALS AND METHODS

### Strains, plasmids, media and reagents

The yeast strain used in this study was *S. cerevisiae* VL6-48 (MATα, *trp1*-Δ1, *ura3*-Δ1, *ade2*-101, *his3*-Δ200, *lys2, met14*) (22). The yeast cells were cultured in YPAD medium (1% [w/v] Bacto yeast extract, 2% [w/v] Bacto peptone, 2% [w/v] glucose, and 80 mg/L adenine hemisulfate) or synthetic complete medium (SC) (Sigma-Aldrich, St. Louis, MO, USA) lacking the nutrients required by the corresponding auxotrophic strains. The *E. coli* strain DH10B (F^−^, λ^−^, *endA1, glnV44, thi-1, recA1, relA1, gyrA96, deoR, nupG, Φ80dlacZ*ΔM15, Δ(*lacZYA-argF*)U169, *hsdR17*(r_K_^−^ m_K_^+^), *mcrA, mcrBC, mrr, galE*) was used for the routine genetic modifications.

The plasmids and primers used in this study are listed in Supplementary Tables S1 and S2. The primers were synthesized by Genescript (Nanjing, Jiangsu, China) or JIE LI Biology (Shanghai, China). Phanta Super-Fidelity DNA Polymerase (Vazyme, Nanjing, Jiangsu, China) and KOD-Plus-Neo DNA Polymerase (Toyobo, Osaka, Japan) were used for the standard PCR amplification. KOD-FX DNA Polymerase (Toyobo, Osaka, Japan) was used for the colony PCR confirmation. The Wizard SV Gel and PCR Clean-up System (Promega, Fitchburg, WI, USA) was used for DNA purification. The restriction endonucleases and DNA size markers were purchased from New England BioLabs (Ipswich, MA, USA). All of the chemicals were obtained from Sigma-Aldrich unless otherwise specified.

### Construction of plasmids

Plasmids p426-SNR52p-gRNA.CAN1.Y-SUP4t and p415-GalL-Cas9-CYC1t were gifts from George Church (15) (Addgene plasmids #43803 and #43804). To construct the plasmid pMetcas9-0 for the constitutive expression of Cas9, the two fragments released by HpaI/SpeI digestion of p415-GalL-Cas9-CYC1t were gel purified. The selection marker *MET14* was PCR amplified from the *S. cerevisiae* S288c genome using the primers Met14-F/Met14-R. The strong constitutive promoter TEF1 was amplified by PCR using the template pAG36 (23) and primers Tef1p-F/Tef1p-R. The PCR primers were designed to contain 40 bp overlapping sequences with the adjacent DNA segments. The four DNA segments described were co-introduced into yeast and assembled into pMetcas9-0. To eliminate the guide RNA expression plasmid, the guide RNA targeting the replication origin of pTrp at the S3 site was synthesized by PCR amplification using the template p426-SNR52p-gRNA.CAN1.Y-SUP4t and primers Cas-S3-F/Cas-S3-R. The galactose inducible GAL1 promoter was PCR amplified from p415-GalL-Cas9-CYC1t using GalL-F/GalL-R. The two DNA segments were assembled by fusion PCR and then cloned into pMetcas9-0 at the XbaI site to construct pMet-Cas9 (Supplementary Figure S1).

To construct the vector backbone pTrp to facilitate the cloning of constitutively expressed guide RNAs, the auxotrophic marker *TRP1* was PCR amplified from the *S. cerevisiae* S288c genome using the primers Trp1-F/Trp1-R. The 2 μm origin, pBR322 origin and ampicillin resistance gene were PCR amplified from p426-SNR52p-gRNA.CAN1.Y-SUP4t using the primer pairs 2μ ori-F/2μ ori-R, pBR322-F/pBR322-R, and f1ori-F/f1ori-R separately. The four DNA fragments were co-introduced into yeast by transformation and then assembled into pTrp. For the first round of DNA assembly, the Target1 and Target2 DNAs, which constitutively express the guide RNAs targeting the common vector backbone of all of the circular DNAs at the S1 (5′-ATAGTGTCACCTAAATAGCT-3′) and S2 (5′-CGTAGCAACCAGGCGTTTAA-3′) sites, were synthesized (for the detailed sequences, please refer to Supplementary Figure S2). The synthesized DNA segments were released from the cloning vectors by BglII/SpeI and SpeI/NcoI digestion and ligated together with the BglII/NcoI digested vector pTrp to construct pTrp-gRNA (Supplementary Figure S2). For the second round of DNA assembly, the DNA segments that targeted the common vector backbone of all of the circular DNAs at the S4 (5′-CGGCCAACGCGAACCCTTAG-3′) and S5 (5′-TGATGAACCTGAATCGCCAG-3′) sites were synthesized and similarly cloned into pTrp to construct pTrp-gRNA2.

The assembly vector pCriv0 (Supplementary Figure S3) was PCR amplified using the primers pCriv0-VF/pCriv0-VR and the DNA template pZQ233 (constructed by our lab, unpublished) to contain the replication origin of chromosome II (24) from *Vibrio cholerae* N16961 (25), the chloramphenicol resistance marker (Cm), and the yeast origin *CEN6 ARS4*. The yeast selection marker *ADE2* was amplified from the *S. cerevisiae* S288c genome using the primers ade2-F/ade2-R. The two DNA fragments were introduced into yeast and then assembled into pCriv0 through 40 bp overlaps at the termini.

The assembly vector pZJ231 (Supplementary Figure S4) for EMG-syn1.0 was constructed by assembling the following four DNA segments. The replication origin of *V. cholerae* chromosome II and its partition system as well as the yeast origin *CEN6 ARS4* and the selection marker *HIS3* were PCR amplified from pZQ233. The kanamycin resistance gene was PCR amplified using pET28 as a template. The 500 bp sequences that overlapped with the large DNA segments Criv7 and TP5 were PCR amplified from the *E. coli* MDS42 genome (26).

### Co-introduction of multiple small or large circular DNAs

The circular DNAs constructed from our previous work (27) were used for the assembly process in this study (Table 1). Two or three small circular DNAs to be assembled were co-introduced into the yeast cells harbouring pMet-Cas9 by using the lithium acetate (LiAc) transformation procedure (28). Circular DNAs larger than 100 kb were co-introduced into the yeast cells by using the protoplast fusion procedure (29,30). Cells harbouring two and three circular DNAs in addition to pMet-Cas9 were selected on the synthetic drop-out media SC-Met-His-Ura (omitting methionine, histidine, and uracil) and SC-Met-His-Ura-Lys (omitting methionine, histidine, uracil, and lysine), respectively. The cells were allowed to grow for 3 days at 30°C until colonies appeared.

**Table 1.** Efficiency of CasHRA in the assembly of both small and large circular DNAs

Circular DNAs joined in assembly		Length of assembled DNA (kb)^a^	Name of assembly	No. of transformants^b^ in three independent experiments	Assembly efficiency^c^
Name	Length of DNA (kb)^a^				
Two small circular DNAs assembly					
pAEEG5	10	25	pCriv4	27, 232, 173	87 ± 15%
pAEEG6	15				
Three small circular DNAs assembly					
pAEEG4	14				
pAEEG5	10	39	pCriv5	77, 244, 116	60 ± 31%
pAEEG6	15				
Two large circular DNAs assembly					
pSP5	117	302	pCriv6	28, 74, 27	80 ± 17%
pTP3-U	185				
Three large circular DNAs assembly					
pTP1	177				
pTP2	298	660	pCriv7	7, 10, 6	73 ± 9%
pTP3-L	185				
Assembly of the 1.03 Mb MGE-syn1.0 (Minimal Genome of *E. coli*)					
pCriv7	660				
pTP4	185	1028	MGE-syn1.0	4, 7, 11	65 ± 13%
pTP5	185				

^a^The DNA length was calculated without the vector backbone.

^b^Transformants of pCriv4–pCriv7 were plated on triple-drop-out medium SC-Met-Trp-Ade (omitting methionine, tryptophan, and adenine) for the selection of the assembly vector (auxotrophic marker ADE2) in addition to the complete Cas9 system. The auxotrophic marker in the MGE-syn1.0 assembly vector was HIS3; thus, the corresponding transformants were plated on triple-drop-out medium SC-Met-Trp-His (omitting methionine, tryptophan, and histidine).

^c^The assembly efficiency was evaluated by PCR positive rates from three independent experiments.

### Assembly booting

The unique linear assembly vector was PCR amplified using pCriv0 as the template and primers that included 60 bp sequences that overlapped the adjacent DNA segments. Approximately 1 μg pTrp-gRNA and 1 μg linear assembly vector (except for the first assembly experiment of pCriv4, for which only half the amount of each DNA was used) were co-introduced into the yeast cells containing circular DNAs to be assembled and pMet-Cas9 by using LiAc transformation. Five percent of the transformants were selected on double-drop-out medium SC-Met-Trp (omitting methionine and tryptophan) for selection of the complete Cas9 system (pMet-Cas9 and pTrp-gRNA). The remaining transformants were plated on the triple-drop-out medium SC-Met-Trp-Ade (omitting methionine, tryptophan, and adenine) for selection of the assembly vector as well as the complete Cas9 system. The transformants were allowed to grow for 2 days at 30°C until colonies appeared.

### MGE-syn1.0 assembly

The guide RNA expression plasmid pTrp-gRNA was eliminated using a previously described protocol (15). After the elimination of pTrp-gRNA, the yeast cells containing pMet-Cas9 (auxotrophic marker Met) and pCriv7 (auxotrophic marker Ade) were protoplast fused with a yeast cell derivative that harboured pTP4 (auxotrophic marker Ura) and a yeast cell derivative that harboured pTP5 (auxotrophic marker Lys), respectively. Cells containing all three large circular DNAs and pMet-Cas9 were selected on SC-Met-Ade-Ura-Lys (omitting methionine, adenine, uracil, and lysine) medium.

The linear assembly vector was generated by PCR amplification using the template pZJ231 and the primers pCriv8-VF/ pCriv8-VR. Approximately 1 μg pTrp-gRNA2 and 1 μg linear assembly vector were co-introduced into the yeast cells by LiAc transformation. Five percent of the transformants were selected on the double-drop-out medium SC-Met-Trp. The remaining transformants were plated on the triple-drop-out medium SC-Met-Trp-His (omitting methionine, tryptophan, and histidine). The transformants were allowed to grow for 2 days at 30°C until colonies appeared.

### Confirmation of the correct assemblies

Correct assemblies were confirmed by colony PCR using the primers listed in Supplementary Table S2. For each assembly, PCR products from five to eight randomly selected positive colonies were sent for sequencing using the same primers used for the PCR assay. The positive assemblies were further subjected to enzymatic digestion and electrophoresis analysis. Small assemblies (pCriv4 and pCriv5) were isolated from the yeast and introduced into *E. coli* DH10B by electroporation. The assemblies re-isolated from *E. coli* were subjected to enzymatic digestion and an electrophoresis analysis. It was difficult to introduce DNAs of several hundred kilobases into *E. coli*; therefore, the large assemblies pCriv6, pCriv7 and MGE-syn1.0 were isolated directly from the yeast and analysed. Approximately 5 × 10^8^ yeast cells were collected and embedded in agarose plugs using a previously described protocol (14). The linear yeast chromosomal DNAs were separated out of the agarose plugs by pulsed-field gel electrophoresis (PFGE) performed under the following conditions: 6 V/cm, 10–60 s switch time, and 14°C for 20 h. The large circular assembly trapped in the agarose plugs was subjected to restriction enzymatic digestion and then a PFGE analysis performed under the following conditions: 6 V/cm, 1–25 s switch time, and 14°C for 20 h.

## RESULTS

### Design of the CasHRA method

**Figure 1.**
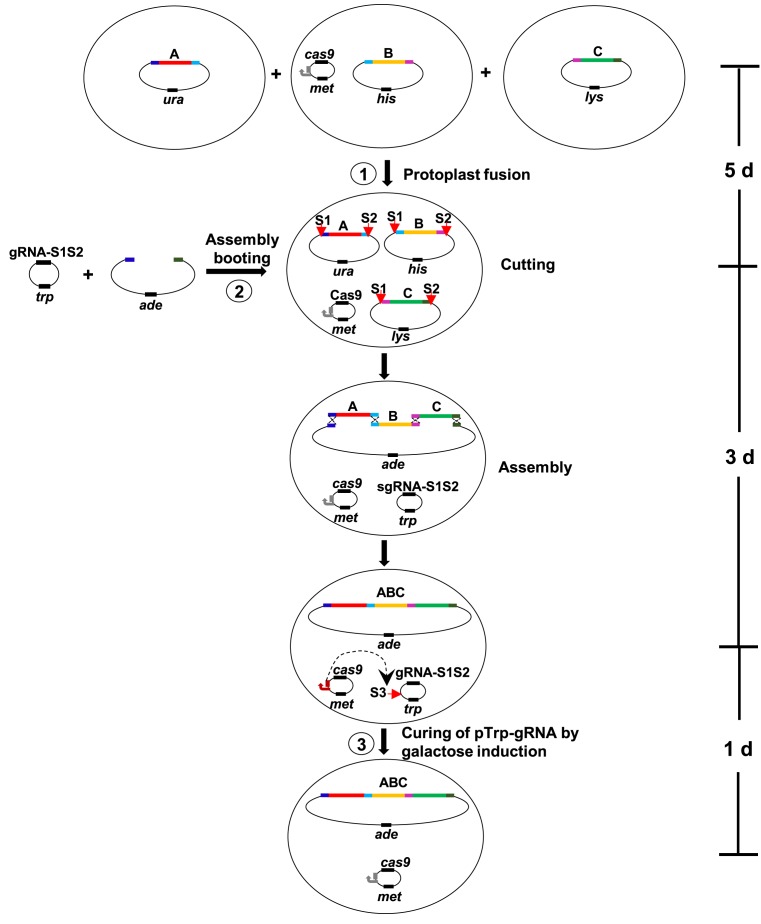
Schematic diagram of the CasHRA method. Large circular DNAs were co-introduced into individual yeast cells harbouring the Cas9 expression plasmid pMet-Cas9 by protoplast fusion (step 1). Then, the guide RNA expression plasmid pTrp-gRNA and the linear assembly vector were co-introduced into the cells by transformation. The RNA-guided Cas9 cut the vector backbone of all of the large circular DNAs at the S1 and S2 sites. The released linear DNA segments were joined together with the linear vector through overlaps by taking advantage of the efficient homologous recombination system in yeast. The plasmid pMet-Cas9 contained a galactose-inducible guide RNA targeting the replication origin of pTrp-gRNA at the S3 site.

CasHRA was developed to facilitate the direct use of large circular DNAs for one-step assemblies *in vivo* and avoid the substantial manipulation of linear DNA segments *in vitro*. As illustrated in Figure 1, three large circular DNAs were co-introduced into yeast cells harbouring the Cas9 expression plasmid pMet-Cas9 via protoplast fusion (step 1). The assembly booting was achieved by co-introducing pTrp-gRNA and the linear assembly vector into the cells by transformation (step 2). The plasmid pTrp-gRNA constitutively expressed guide RNAs that targeted the common vector backbone of all of the large circular DNAs at the S1 and S2 sites. Once pTrp-gRNA entered the cell, a functional Cas9 system rapidly developed and cleaved the large circular DNAs to release the linear DNA segments with exposed overlaps at the termini. Taking advantage of the highly efficient homologous recombination system of *S. cerevisiae*, these large DNA segments were then properly assembled together with the linear assembly vector. The plasmid pMet-Cas9 contained a galactose-inducible guide RNA that targets the replication origin of pTrp-gRNA at the S3 site. Transferring the yeast cells into the galactose medium efficiently eliminated pTrp-gRNA (step 3) and allowed the yeast cells containing the assembled circular DNA to be directly used for the next round of DNA assembly. The entire CasHRA process requires 9 days, including 5 days to introduce all of the circular DNAs into the yeast cells, 3 days to obtain the yeast cells with the assembled product, and 1 day to eliminate pTrp-gRNA.

### Efficiency of CasHRA for circular DNA assembly

We validated the efficiency of CasHRA for the assembly of both small and large circular DNAs as listed in Table 1. These circular DNAs contained 10–298 kb of essential and important growth genes for *E. coli* and were constructed in our previous work (27). The overlaps in the adjacent DNA segments were designed to be approximately 500 bp. Shorter overlap sequences could also be used; however, longer overlaps tended to increase the assembly efficiency (10). The common vector backbone of these circular DNAs contained *CEN6* and *ARS4* for replication and single-copy maintenance in yeast and were used to avoid any discrepant assembly in the yeast (10). Additional yeast replication origins were included in every ∼100 kb of *E. coli* DNA to improve the stability of these large DNA segments in yeast (31). To increase the stability of the large foreign DNAs in the yeast, different auxotrophic selection markers were used to ensure the co-occurrence of the subassemblies in the yeast cells.

To properly validate the success rate, all of the assembly reactions were repeated three times. As shown in Table 1, the success rates of pCriv4 and pCriv5, which were assembled from two and three small circular DNAs, were 87 ± 15% and 60 ± 31%, respectively. Similar efficiencies were obtained for the assembly of circular DNAs larger than 100 kb. The success rates of pCriv6 and pCriv7, which were assembled from two and three large circular DNAs, were 80 ± 17% and 73 ± 9%, respectively. Sequencing 82 overlaps from 23 assembly DNA molecules revealed 10 errors, all of which occurred in the 60 bp overlaps between the vector and the leftmost or rightmost subassemblies. The 60 bp overlaps were introduced by the 3′ ends of the primers during PCR amplification of the assembly vector, and errors could have resulted from impure primers. Detailed information on the enzymatic analysis and sequencing of the assemblies of pCriv4∼7 is presented in Supplementary Figures S5–S8 and Supplementary Table S3.

### Assembly of a 1.03 Mb MGE-syn1.0 genome via CasHRA

**Figure 2.**
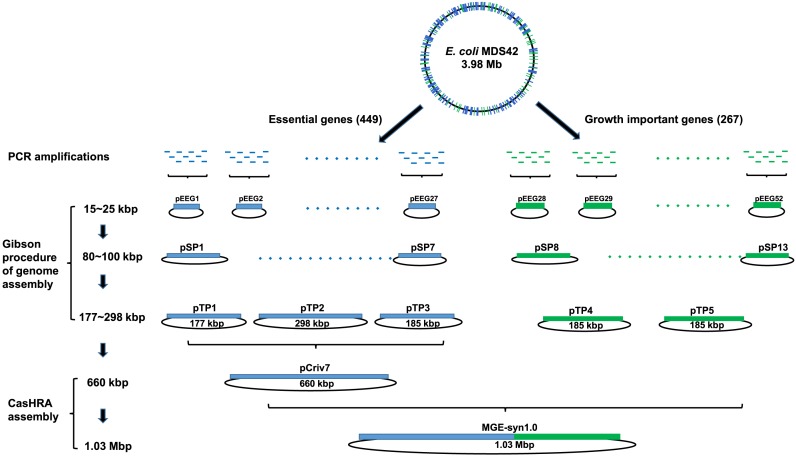
Schematic diagram of the MGE-syn1.0 (Minimal Genome of *E. coli*) assembly. Four hundred forty nine essential genes and 267 important growth genes were PCR amplified from the *E. coli* MDS42 genome, sequenced and assembled into five large, circular DNAs pTP1–pTP5 (177–298 kb *via* three rounds of assembly following the Gibson procedure. The TP1–TP5 were assembled into the 1.03 Mb MGE-syn1.0 via two rounds of CasHRA. Please note that the DNA length was calculated without the vector backbone.

**Figure 3.**
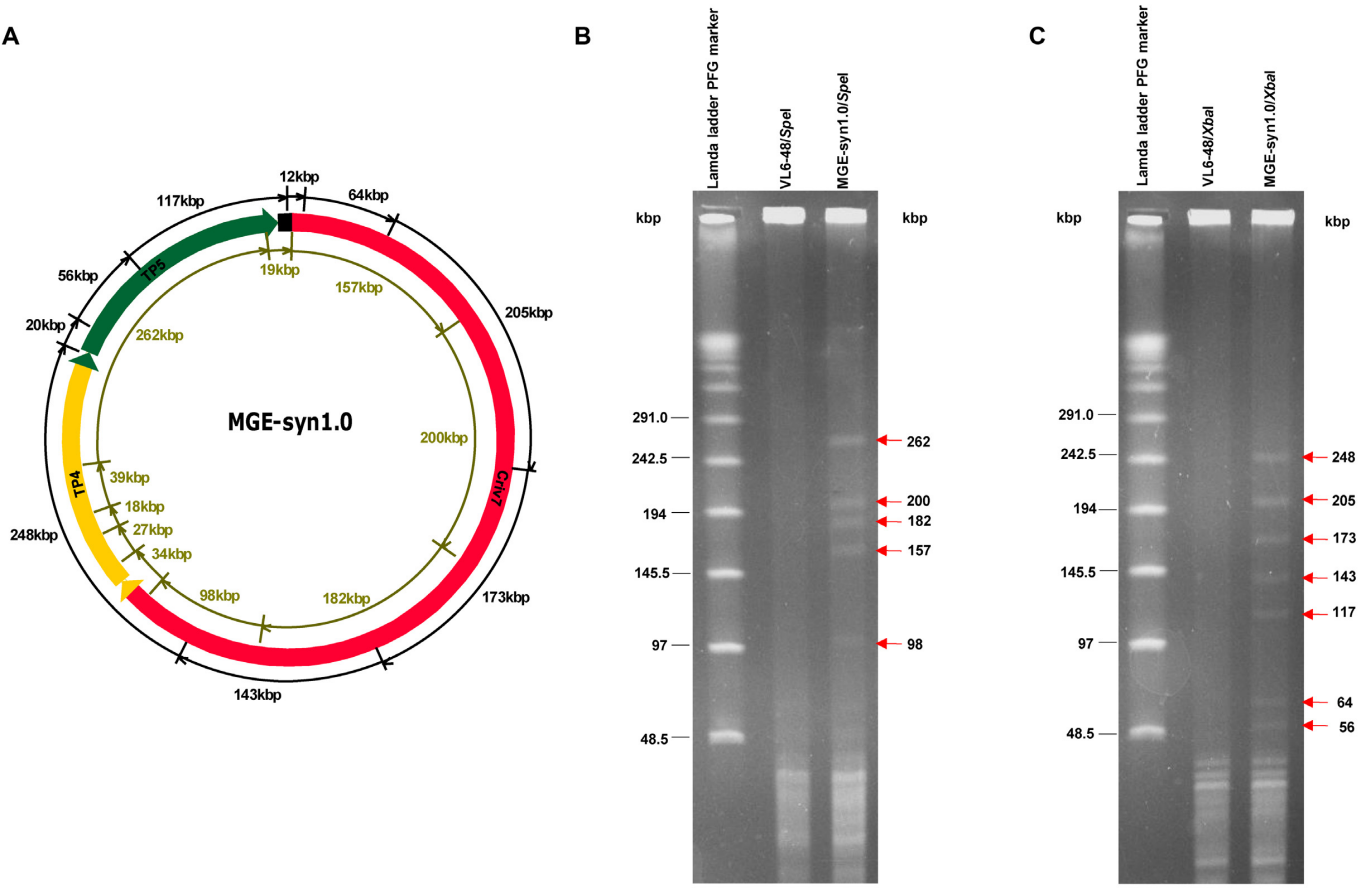
Analysis of the assembled MGE-syn1.0. (**A**) Map of MGE-syn1.0, assembled from three large DNA segments, Criv7 (660 kb, marked in red), TP4 (298 kb, marked in yellow), and TP5 (184 kb, marked in green). The total size of MGE-syn1.0 (1.03 Mb) and the assembly vector (10 kb) was 1.04 Mb. The SpeI cutting sites are marked by greenish brown lines in the inner circle. The XbaI cutting sites are marked by black lines on the outer circle. To separate out the linear yeast chromosomal DNA, agarose plugs were subjected to PFGE under the following conditions: 6 V/cm, 10–60 s switch time, and 14°C for 20 h. The circular MGE-syn1.0 that was trapped inside the plug was digested with SpeI and XbaI, separately, followed by another round of PFGE under the following conditions: 6 V/cm, 1–25 s switch time, and 14°C for 16 h. For the control, the assembly host VL6-48 was subjected to the same treatment. (**B**) The SpeI digestion of MGE-syn1.0 released ten DNA fragments. The five larger DNA fragments with sizes of 262, 200, 182, 157 and 98 kb are indicated by red arrows. Another five smaller bands (39, 34, 27, 19 and 18 kb) could not be clearly separated from the short fragments of yeast chromosomes under the experimental conditions. (**C**) The XbaI digestion of MGE-syn1.0 released nine DNA fragments. The DNA fragments with sizes of 248, 205, 173, 143, 117, 64 and 56 kb are indicated by red arrows. The smaller bands (20 and 11 kb) could not be clearly separated from the yeast chromosomal fragments under the experimental conditions.

To gain a deeper understanding of the basic processes required to create synthetic life, we designed a 1.03 Mb MGE-syn1.0 (Minimal Genome of *E. coli*) that contained 449 essential genes from published experimental and computational studies (27,32–36) and 267 important growth genes, including 151 genes that affect growth (34) and 76 genes that have not been assigned to central metabolism pathways and regulation networks. These genes were PCR amplified from the *E. coli* MDS42 genome (26), sequenced, and then assembled into five large circular DNAs pTP1–pTP5 (Figure 2) by three rounds of assembly following the Gibson procedure (14). The Gibson procedure of genome assembly method can assemble >10 DNA segments at one time with high efficiency; thus, it is a useful method for the early stages of genome assembly. Our previous attempts to sequentially assemble pTP1–pTP3 (177–298 kb) into pCriv7 (660 kb) using the Gibson procedure of genome assembly failed. However, the application of CasHRA successfully assembled pCriv7, which contained all 449 *E. coli* genes. Because of the simplicity and efficiency of CasHRA, we applied this method to assemble the 1.03 Mb MGE-syn1.0. To directly use yeast cells containing pCriv7 for the next round of DNA assembly, the guide RNA expression vector pTrp-gRNA was eliminated by simply transferring the cells into galactose medium, and the elimination frequency was usually >90% in one round of induction. The 1.03 Mb MGE-syn1.0 was assembled from pCriv7 (660 kb), pTP4 (185 kb) and pTP5 (185 kb) (Figure 2). The assembly vector for MGE-syn1.0, pZJ231, was constructed to contain approximately 500 bp overlaps with the left terminus of Criv7 and the right terminus of TP5. The success rate of the MGE-syn1.0 assembly was 65 ± 13% (Table 1), and the sequences of all of the overlaps were 100% correct. The circular MGE-syn1.0 DNA was separated from the yeast linear chromosomes and digested with SpeI and XbaI separately. The digestion patterns were consistent with the theoretical calculation, and DNA bands larger than 50 kb were clearly shown in the pulsed-field gel electrophoresis (PFGE) image (Figure 3), although smaller DNA bands were not separated from the short fragments of the yeast genome under the experimental conditions.

We have attempted to transplant MGE-syn1.0 from yeast to *E. coli* by two different methods to test its functionality. First, we attempted to directly isolate and purify MGE-syn1.0 from yeast and introduce it into *E. coli* DH10B by electroporation. Our pilot study showed that pTP1 (the total size of TP1 and the vector backbone was 187 kb) could be successfully introduced into *E. coli* and stably maintained. However, the introduction of pTP2 (the total size of TP2 and the vector backbone was 308 kb) into *E. coli* failed. The size of TP2 (308 kb) exceeded the maximum size reported for *E. coli* electroporation (37), which might have caused the failure of the TP2 introduction. Second, we attempted to use protoplast fusion to transplant MGE-syn1.0 from yeast to *E. coli*. We prepared the protoplasts of *E. coli* (38) and yeast (29) according to reported protocols and mixed them in different ratios. However, successfully fused *E. coli* cells were not observed in our pilot study. A new method of transplanting giant DNA (>1 Mb) from yeast to *E. coli* awaits further development.

## DISCUSSION

Conventional DNA assembly methods produce purified linear subassembly DNAs by complex manipulations *in vitro*, which can be burdensome in the last stage of megabase-sized DNA (e.g., synthetic genome) assembly. CasHRA was developed to directly use large circular DNAs (>100 kb) in a one-step DNA assembly process *in vivo* that avoids the difficult manipulations *in vitro*. All of the critical steps of CasHRA have been developed *in vivo*, including the co-introduction of large circular DNAs into individual cells by protoplast fusion, the release of large linear DNA segments by RNA-guided Cas9, and the subsequent DNA assembly using the yeast homologous recombination system. The time (9 days) and effort required to assemble DNAs with sizes from hundreds of kilobases (pCriv6 and pCriv7) to one megabase (MGE-syn1.0) were similar. The positive rates of the large assemblies (60–80%) were high enough to allow for easy detection of the correct constructs. Moreover, the efficient elimination of the guide RNA expression vector allowed for the direct use of the yeast cells containing the correct assembly in the next round of DNA assembly, which is convenient for large DNA constructions. Because the activities of CasHRA *in vivo* do not have strict limitations on the size of the subassemblies, we speculate that this method could be used to assemble DNA constructs larger than one megabase.

In our experiments, the subassemblies or the fully assembled molecules could be stably maintained in yeast by selection with different auxotrophic markers. Six auxotrophic markers are available for the *S. cerevisiae* VL6-48 strain. In this study, three auxotrophic markers were replaced by the Cas9 expression plasmid, the gRNA expression plasmid, and the linear assembly vector. The three remaining auxotrophic markers were used for the subassemblies. However, when combined with the commonly used selection markers (e.g. G418, bleomycin and hygromycin), additional subassemblies could be stably maintained in the individual yeast cells. Because of the high performance of Cas9 and the yeast homologous recombination, additional subassemblies could be simultaneously assembled by CasHRA.

To ensure the efficiency of CasHRA, the sequence and length of the assembly overlaps must be considered. In this study, the overlap sequences originated from different regions of the *E. coli* genome (GC content, 51%) to reduce the possible effect of sequence variation in the homology overlaps on the assembly efficiency. Similar efficiencies were obtained for five assembled DNAs with different overlaps, indicating the limited effect of overlap sequence variations on the assembly efficiency in this study. It is important to realize that introducing short overlaps (e.g. in the overlaps between the vector and subassemblies) at the 3′ ends of PCR primers would result in errors in the overlap sequence because of the impurity of the primers. Therefore, pre-cloning the overlaps into the assembly vector will be required if the sequence accuracy of the overlaps are important for the functionality of the assembled DNA. When the assembly efficiencies for both small and large circular DNAs are similar, fewer transformants are obtained from the assembly of large circular DNAs. This result may be caused by a decreased probability of large DNAs finding their counterparts with approximately 500 bp overlaps for homologous recombination. Whether longer overlaps result in more transformants from large DNA assemblies awaits further exploration.

In summary, CasHRA is a promising method for performing the *in vivo* assembly of multiple large DNA segments, and it could be useful in the construction of megabase-sized genomes.

## Supplementary Material

SUPPLEMENTARY DATA
