# Supplementary material for: CasHRA (Cas9-facilitated Homologous Recombination Assembly) method of constructing megabase-sized DNA
Source: Nucleic Acids Res. 2016 May 24;44(14):e124. doi: 10.1093/nar/gkw475 (PMC5001600; doi:10.1093/nar/gkw475)
Supplement: SUPPLEMENTARY DATA [file supp_44_14_e124__index.html]

CasHRA (Cas9-facilitated Homologous Recombination Assembly) method of constructing megabase-sized DNA — SUPPLEMENTARY DATA 

# CasHRA (Cas9-facilitated Homologous Recombination Assembly) method of constructing megabase-sized DNA

## SUPPLEMENTARY DATA

- SUPPLEMENTARY DATA
