# Supplementary material for: CasHRA (Cas9-facilitated Homologous Recombination Assembly) method of constructing megabase-sized DNA
Source: Nucleic Acids Res. 2016 May 24;44(14):e124. doi: 10.1093/nar/gkw475 (PMC5001600; doi:10.1093/nar/gkw475)
Supplement: SUPPLEMENTARY DATA [file supp_gkw475_nar-00500-met-h-2016-File003.pdf]

CasHRA (Cas9-facilitated Homologous Recombination Assembly) to construct megabase-sized DNA

Jianting Zhou, Ronghai Wu, Xiaoli Xue*, Zhongjun Qin*

Supplementary Figure 1:

a

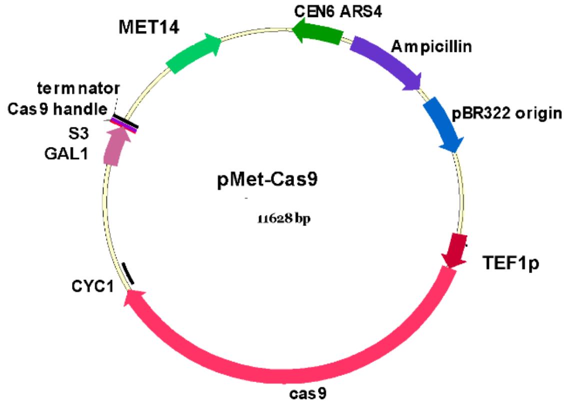

b

target3

GCTCGTGACAGCCCTCCGAAGGAAGACTCTCCTCCGTGCGTCCTCGTCTTC
ACCGGTCGCGTTCCTGAAACGCAGATGTGCCTCGCGCCGCACTGCTCCGAA
CAATAAAGATTCTACAATACTAGCTTTTATGGTTATGAAGAGGAAAAATTGGC
AGTAACCTGGCCCCACAAACCTTCAAATGAACGAATCAAATTAACAACCATA
GGATGATAATGCGATTAGTTTTTTAGCCTTATTTCTGGGGTAATTAATCAGCG
AAGCGATGATTTTTGATCTATTAACGGATATATAAATGCAAAAACTGCATAAC
CACTTTAACTAATACTTTCAACATTTTCGGTTTGTATTACTTCTTATTCAAATGT
AATAAAAGTATCAACAAAAAATTGTTAATATACCTCTATACTTTAACGTCAAGG
AGAAAAAACCCCCTGATTACTAGCGAAGCTGC**GTTTTAGAGCTAGAAATAG**
CAAGTTAAAATAAGGCTAGTCCGTTATCAACTTGAAAAAGTGGCACCGAGT
CGGTGGTGCTTTTTT

Supplementary Figure 1: The Cas9 expression plasmid pMet-Cas9. (a) The schematic map of pMet-Cas9. The yeast replication element (*CEN6 ARS4*) and selection marker (*MET14*, methionine auxotrophic selection) were indicated by green and light green arrowheads separately. The expression of *cas9* (marked by pink) was driven by the strong promoter TEF1p (red color). The *E. coli* replication element (pBR322) and selection marker (ampicillin resistance gene) were shown by blue and violet arrowheads separately. The plasmid pMet-Cas9 contained a galactose inducible guide RNA targeting pTrp replication origin at S3 site. The sequence of target 3 detail was shown in (b). Target 3 consisted GAL1 promoter (marked in rose), base-pairing region for S3 (underlined and marked in red), Cas9 handle (marked in purple), and terminator (black color).

Supplementary Figure 2:

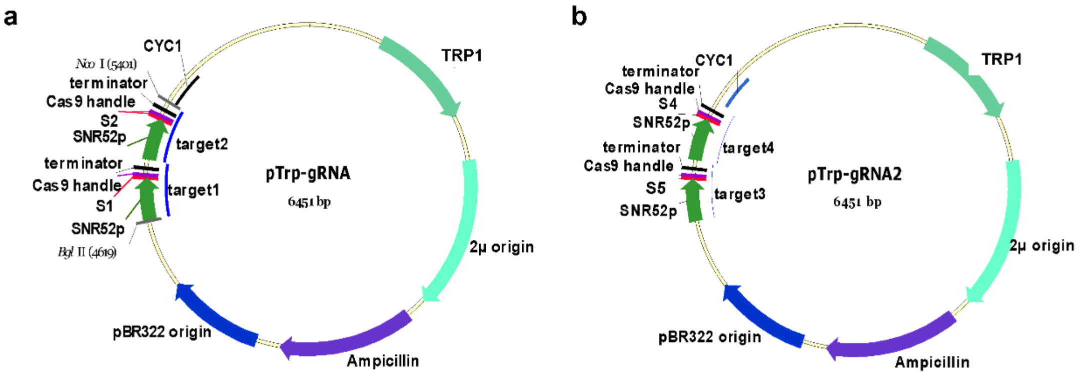

c

target1

ACTTTGAAAAGATAATGTATGATTATGCTTTCACTCATATTTATACAGAAACTTGATGTTTTCTTTCGAGTAT
ATACAAGGTGATTACATGTACGTTTGAAGTACAACTCTAGATTTTGTAGTGCCCTCTTGGGCTAGCGGTAA
AGGTGCGCATTTTTTCACACCCTACAATGTTCTGTTCAAAAGATTTTGGTCAAACGCTGTAGAAGTGAAAG
TTGGTGCGCATGTTTCGGCGTTCGAAACTTCTCCGATAGTGTCACCTAAATAGCTGTTTTAGAGCTAGAAA
TAGCAAGTTAAAATAAGGCTAGTCCGTTATCAACTTGAAAAAGTGGCACCGAGTCGGTGGTGCTTTTTT

target2

ACTTTGAAAAGATAATGTATGATTATGCTTTCACTCATATTTATACAGAAACTTGATGTTTTCTTTCGAGTAT
ATACAAGGTGATTACATGTACGTTTGAAGTACAACTCTAGATTTTGTAGTGCCCTCTTGGGCTAGCGGTAA
AGGTGCGCATTTTTTCACACCCTACAATGTTCTGTTCAAAAGATTTTGGTCAAACGCTGTAGAAGTGAAAG
TTGGTGCGCATGTTTCGGCGTTCGAAACTTCTCCGCGTAGCAACCAGGCGTTTAAGTTTTAGAGCTAGAAA
TAGCAAGTTAAAATAAGGCTAGTCCGTTATCAACTTGAAAAAGTGGCACCGAGTCGGTGGTGCTTTTTT

target4

ACTTTGAAAAGATAATGTATGATTATGCTTTCACTCATATTTATACAGAAACTTGATGTTTTCTTTCGAGTAT
ATACAAGGTGATTACATGTACGTTTGAAGTACAACTCTAGATTTTGTAGTGCCCTCTTGGGCTAGCGGTAA
AGGTGCGCATTTTTTCACACCCTACAATGTTCTGTTCAAAAGATTTTGGTCAAACGCTGTAGAAGTGAAAG
TTGGTGCGCATGTTTCGGCGTTCGAAACTTCTCCGCGGCCAACGCGAACCCTTAGGTTTTAGAGCTAGAA
ATAGCAAGTTAAAATAAGGCTAGTCCGTTATCAACTTGAAAAAGTGGCACCGAGTCGGTGGTGCTTTTTT

target5

ACTTTGAAAAGATAATGTATGATTATGCTTTCACTCATATTTATACAGAAACTTGATGTTTTCTTTCGAGTAT
ATACAAGGTGATTACATGTACGTTTGAAGTACAACTCTAGATTTTGTAGTGCCCTCTTGGGCTAGCGGTAA
AGGTGCGCATTTTTTCACACCCTACAATGTTCTGTTCAAAAGATTTTGGTCAAACGCTGTAGAAGTGAAAG
TTGGTGCGCATGTTTCGGCGTTCGAAACTTCTCCGTGATGAACCTGAATCGCCAGGTTTTAGAGCTAGAAA
TAGCAAGTTAAAATAAGGCTAGTCCGTTATCAACTTGAAAAAGTGGCACCGAGTCGGTGGTGCTTTTTT

Supplementary Figure 2: Targeting guide RNA expression plasmid pTrp-gRNA and pTrp-gRNA2. The vector backbone of pTrp-gRNA and pTrp-gRNA2 were from pTrp, which contained yeast replication element (2 micron origin, allowed high copy number replication in yeast) and selection marker (*TRP1*) were indicated by green and light green arrowheads separately. The pBR322 replication origin and the ampicillin resistance gene were shown by blue and violet

arrowheads separately. The guide RNA expression sequences target 1, 2 and target 4, 5, were synthesized by company and cloned into pTrp to construct pTrp-gRNA (a) and pTrp-gRNA2 (b), respectively. The sequence details of target 1, 2, 4, 5 were shown in (c), which consisted SNR52 promoter (marked in green), base-pairing region for corresponding targeting sites S1, S2, S4, S5 (underlined and marked in red), Cas9 handle (marked in purple), and terminator (black color).

Supplementary Figure 3:

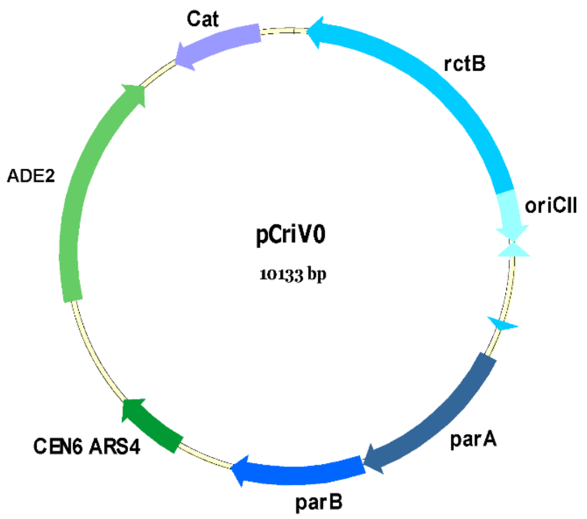

Supplementary Figure 3: The schematic map of the assembly vector pCriv0. The assembly vector pCriv0 contained the replication element (*CEN6/ARS4*), allowing single copy maintenance in yeast, and the selection marker *ADE2*. The origin of *Vibrio cholerae* chromosome II (oriCII) and replication protein coding gene (*rctB*), as well as the partition protein coding genes *parA* and *parB* allowed single copy maintenance in *E. coli*.

Supplementary Figure 4:

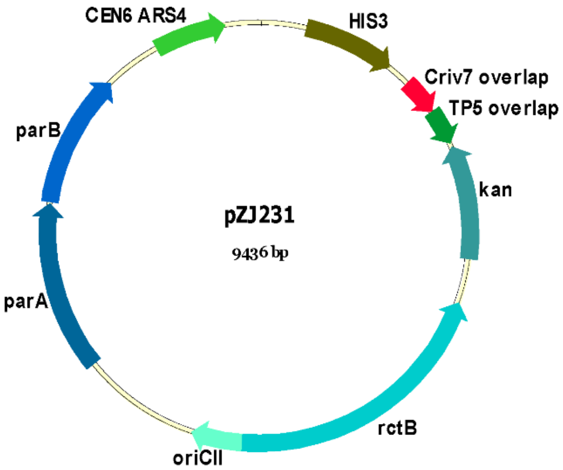

Supplementary Figure 4: The schematic map of the MGE-syn1.0 assembly vector pZJ231. The assembly vector pZJ231 contained the yeast replication element (*CEN6/ARS4*) for single copy maintenance in yeast. The selection marker in yeast is *HIS3*. The origin of *Vibrio cholerae* chromosome II (oriCII) and replication protein coding gene (*rctB*), as well as the partition protein coding genes *parA* and *parB* allowed single copy number maintenance in *E. coli*. The selection marker in *E. coli* is kanamycin resistance gene. The about 500 bp overlaps to Criv7 and TP5 were PCR amplified from *E. coli* MDS42 and cloned in pZJ231.

Supplementary Figure 5:

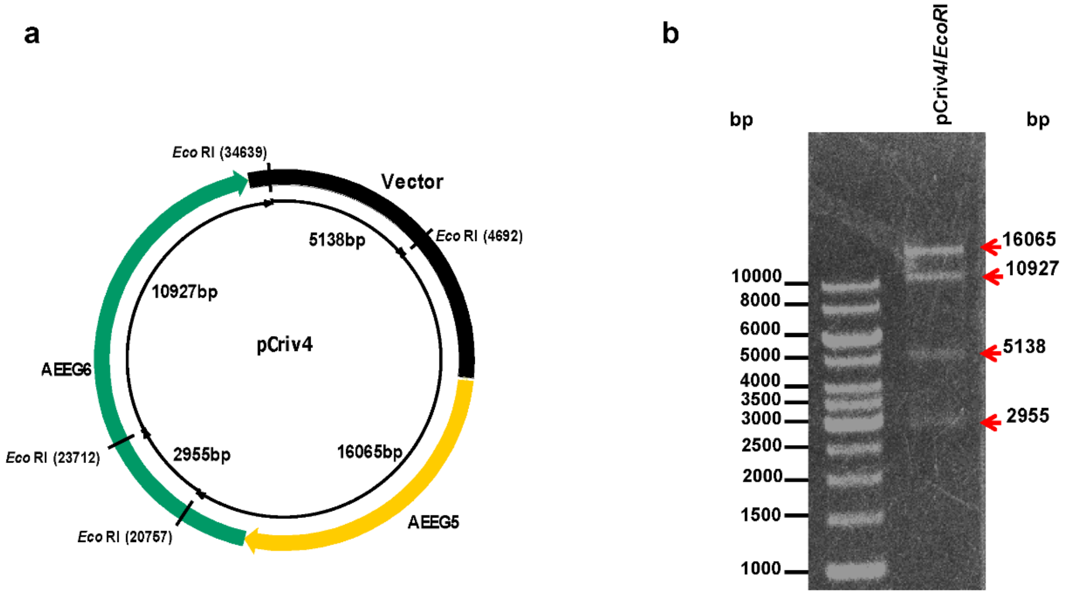

Supplementary Figure 5: Analysis of pCriv4. (a) Map of pCriv4 assembled from two small circular DNAs, which contained AEEG5 and AEEG6. The total size of Criv4 (25 kbp) and the vector (10 kbp) was 35 kbp. (b) The gel electrophoresis analysis of *Eco*RI digested pCriv4, which released five fragments with size of 16065, 10927, 5138, and 2955 bp.

Supplementary Figure 6:

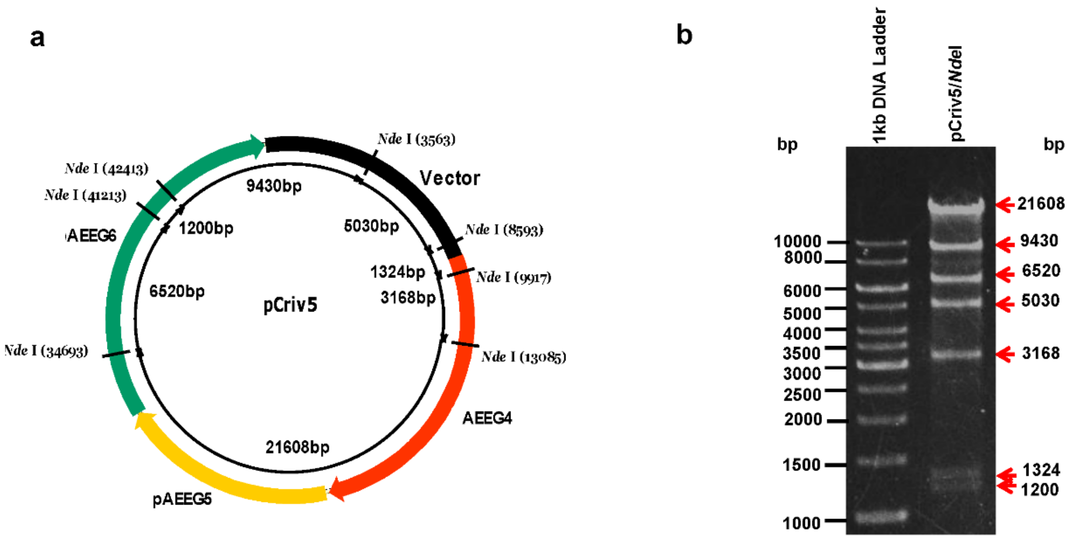

Supplementary Figure 6: Analysis of pCriv5. (a) Map of pCriv5 assembled from three small circular DNAs, which contained AEEG4, AEEG5 and AEEG6. The total size of Criv5 (38 kbp) and the vector (10 kbp) was 48 kbp. (b) The gel electrophoresis analysis of *Nde*I digested pCriv5, which released seven fragments with size of 21608, 9430, 6520, 5030, 3168, 1324, and 1200 bp.

Supplementary Figure 7:

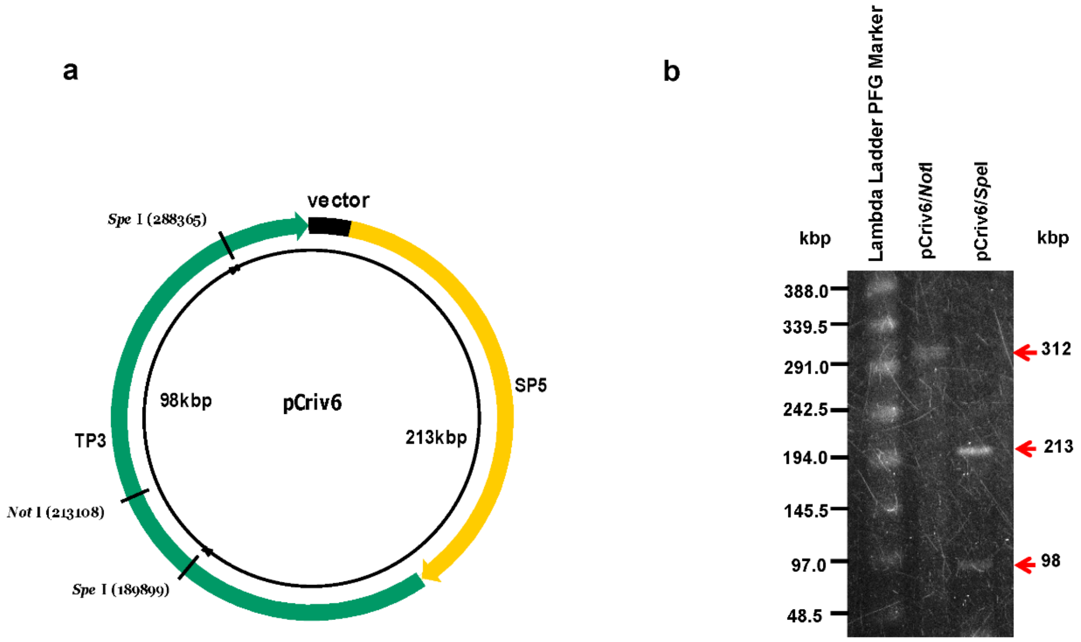

Supplementary Figure 7: Analysis of pCriv6. (a) Map of pCriv6 assembled from two large circular DNAs, which contained SP5 and TP6. The total size of Criv4 (302 kbp) and the vector (10 kbp) was 312 kbp. (b) The pulsed-field gel electrophoresis (PFGE) confirmation of pCriv6. *Not*I digestion of pCriv6 resulted in one 312 kbp linear fragment. *Spe*I digestion of pCriv6 released two fragments with size of 213 and 98 kbp.

Supplementary Figure 8:

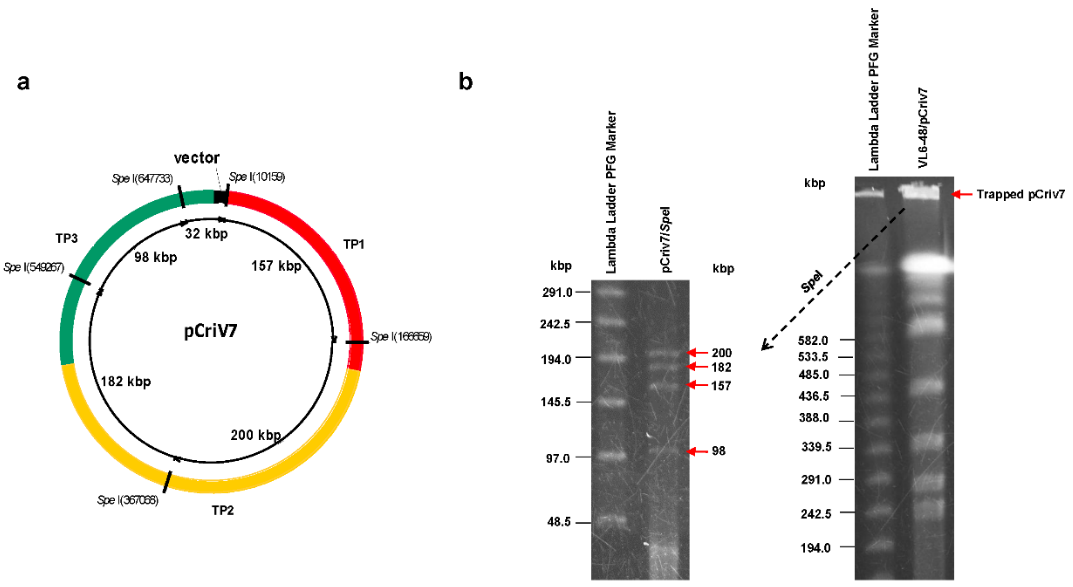

Supplementary Figure 8: Analysis of the assembled pCriv7. (a) Map of pCriv7, assembled from three large circular DNAs, containing TP1 (177 kbp, marked in red), TP2 (298 kbp, marked in yellow), and TP3 (185 kbp, marked in green). The total size of Criv7 (660 kbp) and the assembly vector (10 kbp) was 670 kbp. (b) PFGE analysis of pCriv7. To separate out the linear yeast chromosomal DNA, the yeast agarose plug was subjected to PFGE at 6 V/cm, switch time 10–60 second, 14 ℃ for 20 hours. The circular pCriv7 that trapped inside the plug was subjected to *Spe*I digestion, following by another round of PFGE carried out at 6 V/cm, switch time 1–25 second, 14 ℃ for 20 hours. The *Spe*I digestion of pCriv7 released five fragments with size of 200, 182, 157, 98, and 32 kbp. The smallest band (32 kbp) could not be clearly separated from the short fragments of yeast chromosome under the experiment condition.

Supplementary Table 1: Plasmids used in this study.

**Plasmid name**	**Description**	**Resource**
p415-GalL-hCAS9	p415-GAlL-cas9-SUP4 3'flanking region	Purchased from AddGene
p426-crRNA	p426-SNR52-CAN.Y gRNA-SUP4 3'flanking region	Purchased from AddGene
pAG36	natMax4 *CEN URA3* Tef1p	Goldstein and McCusker 1999
pZQ233	VoriII-Cm-ARS4/CEN6-HIS3	Constructed by our lab
pET28	kanamycin resistance, His-tagged protein expression vector	Preserved in our lab
pMetcas9	p415-GalL-hCAS9：Met14-Tef1p-Cas9-CYC1t	This study
pMet-Cas9	pMetcas9：GAL1p-gRNA-S0-CYC1t	This study
pTrp	p426-crRNA：Trp1-SNR52-CYC1t	This study
pTrp-gRNA	pTRP：sgRNA.target1&2	This study
pTrp-gRNA2	pTRP：sgRNA.target3&4	This study
pCriv0	Ade2-the replication origin of *Vibrio cholerae* chromosome II	This study
pZJ231	VoriII-Kan-ARS4/CEN6-HIS3	This study
pAEEG4	Containing 13700 bp of *Escherichia coli* essential genes	Constructed by our lab
pAEEG5	Containing 9969 bp of *E. coli* essential genes	Constructed by our lab
pAEEG6	Containing 15212 bp of *E. coli* essential genes	Constructed by our lab
pTP1	Containing 177147 bp of *E. coli* essential genes	Constructed by our lab
pTP2	Containing 297952 bp of *E. coli* essential genes	Constructed by our lab
pTP3-L	Containing 184475 bp of *E. coli* essential genes	Constructed by our lab
pTP3-U	Containing 184475 bp of *E. coli* essential genes	Constructed by our lab
pSP5	Containing 116643 bp of *E. coli* essential genes	Constructed by our lab
pTP4	Containing 184794 bp of *E. coli* essential genes	Constructed by our lab
pTP5	Containing 184593 bp of *E. coli* essential genes	Constructed by our lab
pCriv7	Containing 659554 bp of *E. coli* essential genes	This study

Supplementary Table 2: Primers used in this study.

Primer name	Sequence (5′ － 3′)	Description
**For construction of pMet-Cas9**		
Met14-F	AGTAATTGGTTGTTTGGCCGAGCGGTCTAAGGCGCCTGATTCAAGAAATATCTTGACCGCAAGATTACGTTTAAAGGAGCATTAACAG	Forward primer for MET14
Met14-R	TCGTTTCTATTATGAATTTCATTTATAAAGTTTATGTACAAATATCATAAAAAAAGAGAATCTCAAATTACAAATGCTTACGGATGAT	Reverse primer for MET14
Tef1p-F	CTCTATACTTTAACGTCAAGGAGAAAAAACCCCGGATTCTAGCTTGCCTTGTCCCCGCCGGGTCA	Forward primer for Tef1 promotor
Tef1p-R	TTGTGCCGATATCGAGCCCAATGGAGTACTTCTTGTCCATGGTTGTTTATGTTCGGATGTGATG	Reverse primer for Tef1 promotor
GalL-F	GCAGCTTCGCTAGTAATCAGGGGGTTTTTTCTCCTTGACGTTAAAG	Forward primer for GAlL promotor
GalL-R	TGATTCTAGAGCTCGTGACAGCCCTCCGAAGGA	Reverse primer for GAlL promotor
Cas-S3-F	ACTTCTAGAAAAGCCTTCGAGCGTCCCAAAACCT	Forward primer for S3 handle Cas9
Cas-S3-R	CTGATTACTAGCGAAGCTGCGTTTTAGAGCTAGAAATAGCAAGTT	Reverse primer for S3 handle Cas9
**For construction of pTrp**		
Trp1-F	GTTTAGTATACATGCATTTACTTATAATACAGTTTTTCGCGCGTTTCGGTGATGACGGT	Forward primer for TRP1 with f1 origin overlap
Trp1-R	ATAACTTCGTATAATGTATGCTATACGAAGTTATGATCGGCAAGTGCACAAACAATAC	Reverse primer for TRP1 with 5'loxp overlap
2μ ori-F	ATAACTTCGTATAGCATACATTATACGAAGTTATCAGGAGGTACTAGACTACCTTTCAT	Forward primer for 2u origin with 5'loxp overlap
2μ ori-R	ATAACTTCGTATAATGTATGCTATACGAAGTTATAAGTGCCACCTGAACGAAGCATCTG	Reverse primer for 2u origin with 3'loxp overlap
pBR322-F	CTTATAACTTCGTATAGCATACATTATACGAAGTTATTTCGGGGAAATGTGCGCGGAAC	Forward primer for pBR322 with 3'loxp overlap
pBR322-R	AATTACATGACTCGACCATGGCCTAGGAGATCTAGCTCCAGCTTTTGTTCCCTTTAGTG	Reverse primer for pBR322 with f1 origin overlap
f1ori-F	GCTGGAGCTAGATCTCCTAGGCCATGGTCGAGTCATGTAATTAGTTATGTCACGCTTAC	Forward primer for f1 origin with pBR322 overlap
f1ori-R	GAGGTTTTCACCGTCATCACCGAAACGCGCGAAAAACTGTATTATAAGTAAATGCATGT	Reverse primer for f1 origin with TRP1 overlap
**For construction of pCriv0**		

pCriv0-VF	CCAACGCGAACCGCGGCCGCGGCGTTTAAGGGCACCAATAACTGC	Forward primer for the replication origin of *V.* cholerae chromosome II with ADE2 overlap
pCriv0-VR	GATGATATACTGGTAGTGCGCTGTGGTATGGTGCACTCTCAGTAC	Reverse primer for the replication origin of *V. cholerae* chromosome II with ADE2 overlap
ade2-F	GAGAGTGCACCATACCACAGCGCACTACCAGTATATCATCTCATTTC	Forward primer for ADE2 with the replication origin of V. cholerae chromosome II overlap
ade2-R	CCCTTAAACGCCGCGGCCGCGGTTCGCGTTGGCCGATTCATTAAT	Reverse primer for ADE2 with the replication origin of V. cholerae chromosome II overlap
**For construction of pZJ231**		
ZJ231-F	GCAAGACGTTTCCCGTTGAATATGGCTCATTTTAGCTTCCTTAGCTCCTGAAAATCTCG	Forward primer for oriCII and CEN6 ARS4
ZJ231-R	CCACTAAGCCAAGCTATTTAGGTGACACTATCTTCAGAATGACACGTATAGAATGATGC	Reverse primer for oriCII and CEN6 ARS4
Criv7-ov-F	CATTCTGAAGATAGTGTCACCTAAATAGCTTGGCTTAGTGGGAAGAGGTAGGGGGATG	Forward primer for Criv7 overlap
Criv7-ov-R	CAATTTCATGTCTGTGACGCGACTCACTCACTGCGGTTGACTACT	Reverse primer for Criv7 overlap
TP5-ov-F	TCAACCGCAGTGAGTGAGTCGCGTCACAGACATGAAATTGGTAAG	Forward primer for TP5 overlap
TP5-ov-R	AGAATTAACCCTTAAACGCCTGGTTGCTACGCGACGAAGCAACAAGCCCTTC	Reverse primer for TP5 overlap
kan-F	CGCGTAGCAACCAGGCGTTTAAGGGTTAATTCTTAGAAAAACTCATCGAGCATC	Forward primer for kanamycin resistant gene
kan-R	GTTATCGAGATTTTCAGGAGCTAAGGAAGCTAAAATGAGCCATATTCAACGGGAAACG	Reverse primer for kanamycin resistant gene
**For generation of unique linear assembly vector**		
pCriv4-VF	ACGCATAACCATAGCGAAAATAGTGGCGCAGTGTAAGGTTGTTGTGAATATTGAGTTGCAGGCGTTTAAGGGCACCAATAACTGC	Forward primer for linearized pCriv4 vector with EEG6 overlap
pCriv4-VR	GCAATCGGCGAGCTACGCCAACCTGTCTGGCCTGCGGAGGTATACGGCAGTATTAGCCACGGTTCGCGTTGGCCGATTCATTAAT	Reverse primer for linearized pCriv4 vector with EEG5 overlap
pCriv5-VF	CTTACGCATAACCATAGCGAAAATAGTGGCGCAGTGTAAGGTTGTTGTGAATATTGAGTTGCAGGCGTTTAAGGGCACCAATAACTGC	Forward primer for linearized pCriv5 vector with EEG6 overlap

pCriv5-VR	GGAGATAAAATCCCCCCTTTTTGGTTAACTAATTGTATGGGAATGGTTAATTATTCACCAGATGGTTCGCGTTGGCCGATTCATTAAT	Reverse primer for linearized pCriv5 vector with EEG4 overlap
pCriv6-VF	CCCGCCACGAAATTGAGGATGCTAAGGCTGTTGTAGACCGGAAACGGTGTTCACGCCGCATCCGGCGTTTAAGGGCACCAATAACTGC	Forward primer for linearized pCriv6 vector with TP3 overlap
pCriv6-VR	ATCCAGGTACTATGAGCCCAATCCAACACGGGGAAGTGTTCGTTACTGAAGACGGCGCTGAAAGGTTCGCGTTGGCCGATTCATTAAT	Reverse primer for linearized pCriv6 vector with SP5 overlap
pCriv7-VF	GCCACGAAATTGAGGATGCTAAGGCTGTTGTAGACCGGAAACGGTGTTCACGCCGCATCCGGCGTTTAAGGGCACCAATAACTGC	Forward primer for linearized pCriv7 vector with TP3 overlap
pCriv7-VR	ATCTTCTCTTTATCAGCAGTAAACTAGTGGGTATTCATCCCCCTACCTCTTCCCACTAAGGGTTCGCGTTGGCCGATTCATTAAT	Reverse primer for linearized pCriv7 vector with TP1 overlap
pCriv8-VF	TGTGATTGAGCAGCAACTGGAG	Forward primer for linearized assembly vector for MGE-syn1.0
pCriv8-VR	GCGATATGGCAGGACAAGACGT	Reverse primer for linearized assembly vector for MGE-syn1.0
**For verification of assemblies**		
EEG5-T1	GCTACAAATGTAGCGTTGAGGTG	Forward primer for verifying pCriv4 and pCriv5
EEG6-T1	CATCATCACGCTGGGTAAAGG	Reverse primer for verifying pCriv4 and pCriv5
EEG4-T3	AGTCGCTCGTGTTCCGCT	Forward primer for verifying pCriv5
EEG5-T2	CTGTGGATAGCCGCCAGAG	Reverse primer for verifying pCriv5
pCriv6-T2	CTCAAACAGATCATCGTC	Forward primer for verifying pCriv6 and pCriv7
pCriv6-T3	AAGAGCAAGATAAAAGGTA	Reverse primer for verifying pCriv6 and pCriv7
pCriv7-T2	CGCTGGATTTGTCACCTTC	Forward primer for verifying pCriv7
pCriv7-T3	CGATTTGCCAGTTGTTCCAG	Reverse primer for verifying pCriv7
pCriv8-T1	CGCATTGCAGTATTCTGACTACG	Forward primer for verifying MGE-syn1.0
pCriv8-T2	CAATGTTGAACGCGGATCG	Reverse primer for verifying MGE-syn1.0
pCriv8-T3	GAGTCAGAGAGTTAAAGCGAACTGC	Forward primer for verifying MGE-syn1.0

pCriv8-T4	CAACACCCTGACGCGTAAG	Reverse primer for verifying MGE-syn1.0

Supplementary Table 3: Sequencing results of assemblies overlaps.

Name of assembly	Overlaps	colony 1	colony 2	colony 3	colony 4	colony 5	colony 6	colony 7	colony 8
pCriv4	Overlap 1 (vector-AEEG5)	one 13-bp insertion	correct	one 13-bp insertion	correct	correct			
	Overlap 2 (AEEG5-AEEG6)	correct	correct	correct	correct	correct			
	Overlap 3 (AEEG6-vector)	three 1-bp deletions	correct	correct	correct	correct			
pCriv5	Overlap 1 (vector-AEEG4)	correct	correct	correct	correct	two 1-bp deletion			
	Overlap 2 (AEEG4-AEEG5)	correct	correct	correct	correct	correct			
	Overlap 2 (AEEG5-AEEG6)	correct	correct	correct	correct	correct			
	Overlap 3 (AEEG6-vector)	correct	correct	correct	correct	one 1-bp deletion			
pCriv6	Overlap 1 (vector-SP5)	correct	correct	one 1-bp deletion	correct	correct			
	Overlap 2 (SP5-TP3)	correct	correct	correct	correct	correct			
	Overlap 3 (TP3-vector)	correct	correct	one 1-bp deletion	correct	correct			
pCriv7	Overlap 1 (vector-TP1)	correct	correct	correct	correct	correct	one 16-bp insertion	one 16-bp insertion	correct
	Overlap 2 (TP1-TP2)	correct	correct	correct	correct	correct	correct	correct	correct

	Overlap 2 (TP2-TP3)	correct	correct	correct	correct	correct	correct	correct	correct
	Overlap 3 (TP3-vector)	correct	correct	correct	correct	correct	one 1-bp mutation	correct	correct
MGE-syn1.0	Overlap 1 (vector-Criv7)	correct	correct	correct	correct	correct			
	Overlap 2 (Criv7-TP4)	correct	correct	correct	correct	correct			
	Overlap 2 (TP4-TP5)	correct	correct	correct	correct	correct			
	Overlap 3 (TP5-vector)	correct	correct	correct	correct	correct			
